# Restrictive strategy of intraoperative fluid maintenance during optimization of oxygen delivery decreases major complications after high-risk surgery

**DOI:** 10.1186/cc10466

**Published:** 2011-09-23

**Authors:** Suzana M Lobo, Luis S Ronchi, Neymar E Oliveira, Paulo G Brandão, Adriano Froes, Geni S Cunrath, Kátia G Nishiyama, João G Netinho, Francisco R Lobo

**Affiliations:** 1 Division of Intensive Care, Department of Internal Medicine, Faculdade de Medicina de São José do Rio Preto, Av Faria Lima-5544, São José do Rio Preto, CEP-15090-000, Brazil; 2 Division of Colorectal Surgery, Department of Surgery, Faculdade de Medicina de São José do Rio Preto, Av Faria Lima-5544, São José do Rio Preto, CEP-15090-000, Brazil; 3 Department of Anesthesiology, Faculdade de Medicina de São José do Rio Preto, Av Faria Lima-5544, São José do Rio Preto, CEP-15090-000, Brazil

## Abstract

**Introduction:**

Optimal fluid management is crucial for patients who undergo major and prolonged surgery. Persistent hypovolemia is associated with complications, but fluid overload is also harmful. We evaluated the effects of a restrictive versus conventional strategy of crystalloid administration during goal-directed therapy in high-risk surgical patients.

**Methods:**

We conducted a prospective, randomized, controlled study of high-risk patients undergoing major surgery. For fluid maintenance during surgery, the restrictive group received 4 ml/kg/hour and the conventional group received 12 ml/kg/hour of Ringer's lactate solution. A minimally invasive technique (the LiDCO monitoring system) was used to continuously monitor stroke volume and oxygen delivery index (DO_2_I) in both groups. Dobutamine was administered as necessary, and fluid challenges were used to test fluid responsiveness to achieve the best possible DO_2_I during surgery and for 8 hours postoperatively.

**Results:**

Eighty-eight patients were included. The patients' median age was 69 years. The conventional treatment group received a significantly greater amount of lactated Ringer's solution (mean ± standard deviation (SD): 4, 335 ± 1, 546 ml) than the restrictive group (mean ± SD: 2, 301 ± 1, 064 ml) (*P *< 0.001). Temporal patterns of DO_2_I were similar between the two groups. The restrictive group had a 52% lower rate of major postoperative complications than the conventional group (20.0% vs 41.9%, relative risk = 0.48, 95% confidence interval = 0.24 to 0.94; *P *= 0.046).

**Conclusions:**

A restrictive strategy of fluid maintenance during optimization of oxygen delivery reduces major complications in older patients with coexistent pathologies who undergo major surgery.

**Trial registration:**

ISRCTN: ISRCTN94984995

## Introduction

Despite unquestionable advances in the field of perioperative care, morbidity and mortality are still high in some groups of patients, particularly in elderly patients with coexisting pathologies or low cardiorespiratory reserve who undergo extensive and traumatic noncardiac surgery [1,2]. Multiple organ dysfunction and sepsis are complications frequently seen after surgery in such patients [3,4].

Global perfusion is usually assessed by calculation of the oxygen delivery index (DO_2_I). Imbalance between oxygen delivery (DO_2_) and oxygen consumption (VO_2_) can quickly result in irreversible damage to the cells [5]. Perioperative alterations in DO_2 _are closely correlated to the development of multiple organ failure (MOF) and death [6]. The fundamental principle underpinning goal-directed therapy (GDT), better known in the field of perioperative care as "hemodynamic optimization, " is the optimization of tissue perfusion by manipulating stroke volume (SV), hemoglobin and arterial oxygen saturation (SaO_2_) to improve DO_2 _by using fluids, inotropes and red blood cells.

Several randomized clinical trials and meta-analyses have shown reductions in postoperative complications and mortality rates when preemptive strategies aimed at optimizing tissue perfusion were used in the perioperative period [6-21]. In the past, cardiac output (CO) and DO_2 _measurements and GDT were performed by means of pulmonary artery catheters [6-10]. Less invasive methods are now available. The esophageal Doppler-derived corrected flow time is the parameter used most frequently [14-18]. Other measurements, such as SV and CO, obtained by pulse contour analysis with a lithium indicator dilution and pulse power analysis conducted using the LiDCO plus system (Cambridge, UK) and a Vigileo monitor (Edwards Lifesciences, Irvine, CA, USA) or continuous hemodynamic monitoring with a PiCCO monitor (Philips Healthcare, Andover, MA, USA) have been incorporated into clinical practice. High-risk surgical patients allocated to postoperative GDT to attain DO_2_I of 600 ml/minute/m^2 ^measured using the LiDCO system had fewer complications and shorter hospital stays [19].

Perioperative fluid management is a challenge for anesthesiologists and intensivists. The optimal perioperative fluid regimen for major noncardiac surgery is unclear. Some authors have suggested that estimates of ongoing fluid loss that needs to be replaced with crystalloid are certainly excessive [22], and a growing body of evidence demonstrates better outcomes associated with more restrictive fluid replacement strategies [22-29]. These studies were performed using basic monitoring of vital signs, central venous pressure, urine output and body weight. However, high-risk patients who have more traumatic surgery are at a high risk of hypovolemia and cardiac dysfunction, which cannot be detected without more complex monitoring. Moreover, these patients may benefit from GDT. Therefore, in this study of high-risk surgical patients, we evaluated the impact of a restrictive strategy of fluid maintenance compared to a conventional regimen on the incidence of major postoperative complications during GDT aimed at optimizing DO_2_I and reducing serum lactate levels. We also assessed the hemodynamic and perfusion patterns associated with the two regimens.

## Materials and methods

This prospective, randomized, controlled study was approved by the Institutional Review Board at our institution and carried out in the operating room (OR) and 24-bed ICU of a tertiary hospital. This study is registered as ISRCT N94984995. Informed consent was obtained from each patient. Recruitment was interrupted for one year because of lack of funding.

Patients undergoing elective surgery were admitted to the study if their total risk score based a system adapted from the American College of Cardiology/American Heart Association guidelines was ≥3 points [30]. Two points were given for high-risk surgery, one point for intermediate-risk surgery and one point for each clinical predictor (Table 1). Exclusion criteria were refusal of consent, unplanned surgery, unavailability of ICU beds, pregnancy, congestive heart failure (New York Heart Association functional class IV), chronic renal failure (preoperative creatinine > 2.0 mg/dl or need for dialysis), acute myocardial ischemia prior to enrollment (acute myocardial infarction within six months, evidence of a risk of ischemia based on clinical symptoms or findings of noninvasive tests), lithium therapy, or severe ventricular or supraventricular arrhythmia), life expectancy < 60 days and palliative treatment. The presence of one of two trained anesthesiologists or intensivists trained in the study procedures was mandatory, and patients were not enrolled if this condition was not met.

**Table 1:** Risk scoring system (adapted from American College of Cardiology/American Heart Association guidelines)

Risk category	Points assigned
High-risk surgery	
Gastrectomy	2
Pancreatectomy	2
Total colectomy	2
Total esophagectomy	2
Other long surgical procedure associated with large-volume fluid shift or blood loss	2
Intermediate-risk surgery	
Endarterectomy	1
Head and neck	1
Intraperitonial or intrathoracic	1
Orthopedic	1
Clinical predictor	
Age > 60 years old	1
Diabetes (defined as standard taking medication or not)	1
Abnormal electrocardiogram (left ventricular hypertrophy, left bundle branch block, ST-T abnormalities and atrial fibrillation)	1
Low functional capacity (inability to climb one flight of stairs with a bag of groceries)	1
Arrhythmia (receiving drug therapy)	1
History of stroke	1
Arterial hypertension (difficult to control)	1
Compensated or previous decompensated heart failure (defined as standard)	1
Angina (Canadian Cardiovascular Society classification system class I or II) or previous infarct or Q waves, severe valvopathy (severe valvular regurgitation with reduced left ventricular function)	1
Chronic hepatic failure (defined as standard)	1
Chronic renal failure (preoperative creatinine > 2.0 mg/dl or need for dialysis)	1
Chronic obstructive pulmonary disease (defined as standard) or severe respiratory illness resulting in functional limitation	1

Patients undergoing elective surgery were admitted to the study if their total score was ≥3 points based on a risk-scoring system adapted from the American College of Cardiology/American Heart Association guidelines [30].

### Treatment algorithm

After admission and in the OR, an oxygen catheter (2 L/minute), a central venous catheter and an arterial catheter for measurement of mean arterial pressure (MAP) were immediately placed, and samples were taken for baseline blood tests. Measurements of hematocrit, sodium, arterial and venous blood gases, and serum lactate were obtained hourly during surgery, every 2 hours for 8 hours in the ICU and 24 hours after ICU admission. Serum C-reactive protein (CRP) and creatinine were measured after admission to the ICU and 24 hours afterward. Electrocardiogram (EKG), pulse oximeter and MAP readings were monitored continuously during the study period. Acid-base and hydroelectrolytic disturbances were corrected according to routine procedures.

A minimally invasive technique utilizing the LiDCO™plus Hemodynamic Monitor system with lithium dilution was applied to continuously monitor SV, CO, DO_2_I and systemic vascular resistance index. DO_2_I values, SV, serum lactate and central venous oxygen saturation (S_CV_O_2_) were recorded hourly. A bolus of 10 ml/kg of 0.9% saline was administered before the induction of anesthesia in patients in the two groups. The patients were then randomized, using sealed envelopes (in blocks of 10), to one of the two groups: restrictive or conventional fluid maintenance. The surgeons were blinded to the treatment assignments. The conventional group received 12 ml/kg/hour of lactated Ringer's solution as maintenance fluid during surgery, and the restrictive group received 4 ml/kg/hour of the same solution.

An epidural catheter was inserted before surgery, and 10 ml of ropivacaine hydrochloride (0.25%) was delivered for postoperative pain relief. To induce anesthesia, the following drugs were used: midazolam 0.05 to 0.10 mg/kg, propofol 2 mg/kg, sufentanil 0.3 to 0.5 μg/kg and atracurium besylate 0.5 mg/kg. Fluid maintenance was achieved by using a balanced technique involving isoflurane, nitrous oxide and oxygen, as well as continuous infusion of sufentanil (0.3 to 0.5 μg/kg/hour) and atracurium (7 to 10 μg/kg/minute).

**Figure 1:**
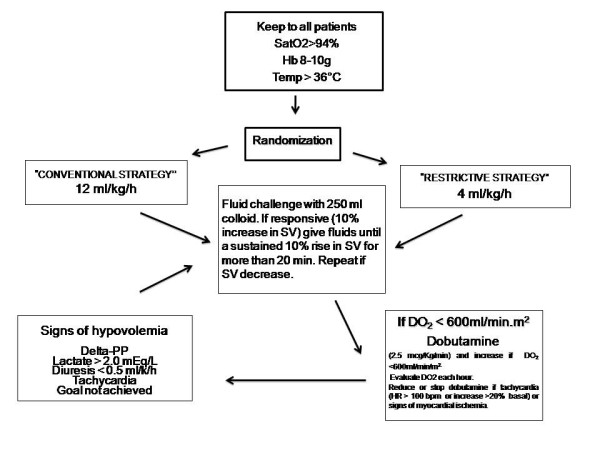
**Algorithm of treatment**. Delta-PP = change in arterial pulse pressure variation; DO_2 _= oxygen delivery; Hb = hemoglobin; SatO_2 _= oxygen saturation; SV = stroke volume.

The treatment algorithm is shown in Figure 1. The therapeutic goals for both groups were to keep hemoglobin between 8 to 10 mg/dl, SaO_2 _> 94%, urine output > 0.5 ml/kg/hour, core temperature > 36°C and DO_2_I as close as possible to or > 600 ml/minute/m^2 ^by using dobutamine beginning at 2.5 μg/kg/minute and progressively increasing until the goal was reached. Dobutamine administration was interrupted in the presence of predefined adverse events such as persistent tachycardia (an increase by 20% from baseline), hypotension unresponsive to fluid challenge, angina and/or signs of myocardial ischemia on the EKG.

Fluid responsiveness was tested after the induction of anesthesia and whenever serum lactate increased to > 2.0 mEq/L for two consecutive measurements, diuresis declined to < 0.5 ml/kg/hour for 2 hours or arterial pulse pressure variation was ≥13%. Fluid responsiveness was evaluated by assessing the response of SV to the infusion of 250 ml of gelatin over 20 minutes. If SV increased by > 10%, fluid replacement was maintained to keep the SV at the achieved value for ≥10 minutes.

In the ICU, both groups received 1.5 ml/kg/hour of lactated Ringer's solution as fluid maintenance. The therapeutic goals in the ICU were the same as in the OR. The same treatment algorithm was used for 8 hours postoperatively. After this period, dobutamine was tapered and then discontinued. In the ICU, the Physiological and Operative Severity Score for the enUmeration of Mortality and morbidity (POSSUM) and respective estimated mortality rates were calculated [31]. The patient was defined as an achiever a DO_2_I > 600 ml/minute/m^2 ^was attained at at least one time point in the period between the start of surgery and 8 hours after ICU admission.

### Outcomes

Patients were followed until hospital discharge or for 60 days. An investigator who was unaware of patient group allocation evaluated complications retrospectively by analyzing the medical records and all radiological images and laboratory tests. Major complications were defined as any untoward medical event that prolonged hospitalization, was life-threatening or caused death.

Anastomotic leakage and wound dehiscence were considered complications of tissue healing. Cardiac arrhythmias with hemodynamic repercussions, need for antiarrhythmic drugs and arterial hypertension that required sodium nitroprusside for control and/or prolonged ICU stay were considered cardiovascular complications. Severe sepsis and septic shock were defined according to the American College of Chest Physicians/Society of Critical Care Medicine guidelines [32]. Centers for Disease Control and Prevention definitions were used to diagnose nosocomial infections. Pulmonary emboli were confirmed by spiral computed tomography. Renal dysfunction was defined as an increase in serum creatinine level by more than twice the baseline level during the postoperative period in patients with previously normal renal function. Gastrointestinal dysfunction was defined as feeding intolerance for more than five days after the operation or the need for parenteral nutrition. Extubation failure was defined as failure to extubate within the first 24 hours after the operation or the need for reintubation within 72 hours after extubation.

### Statistical analysis

The size of the sample was determined based on previous data from our institution showing a morbidity rate of 56% in patients who received GDT during surgery [33]. To achieve a study power of 80% and a two-sided test with a significance of 0.05, 56 patients in each group were required in order to decrease the morbidity rate by 46%. The first statistical evaluation, in which we looked for differences in primary outcomes, was scheduled for when 50% of the planned number of patients were enrolled. At this point, 29 patients were included in the conventional group and 27 were in the restrictive group. At the time of this evaluation, a 50% decrease in the rate of major complications was noted, and a second interim analysis was planned for when around 80% of the original planned population was enrolled. At that point, statistically significant differences in major outcomes were identified and the study was terminated.

Continuous variables are presented as means ± standard deviations or medians with 25% to 75% interquartile ranges, and categorical variables are given as numbers and percentages unless otherwise indicated. The Kolmogorov-Smirnov test was used to verify the normality of distribution of continuous variables. Difference testing between groups was performed using a two-tailed *t*-test and Fisher's exact test as appropriate. Analysis of variance was used for repeated measurements. When there were statistically significant differences, the Bonferroni test was used to detect at which time the differences occurred. The incidence of complications and mortality rates were evaluated using relative risk (RR) (95% confidence interval (CI)). *P *< 0.05 was considered statistically significant.

## Results

A total of 116 patients were evaluated for inclusion in the study between February 2006 and July 2010. Eighty-eight patients were randomized, 43 to the conventional group and 45 to the restrictive group. Twenty-eight patients were excluded because of the unavailability of a trained anesthesiologist or intensivist (*n *= 25) or an ICU bed (*n *= 1), acute myocardial infarction within six months of study inclusion (*n *= 1) and arrhythmia (*n *= 1). Because of technical problems, SV and DO_2 _data were not available for three patients (all in the restrictive group) in the postoperative period (arrhythmia in one patient and loss of an arterial line in two). Patients were included in the analysis on an intention-to-treat basis.

Demographic and risk-scoring data from both groups are shown in Table 2. No significant differences between groups were found. The median age of the patients was 69 years. Pre-, intra- and postoperative data, therapeutic interventions, and tissue perfusion variables are given in Table 3. The conventional group received a significantly greater amount of lactated Ringer's solution (4, 335 ± 1, 546 ml) during surgery than the restrictive group (2, 301 ± 1, 064 ml) (Mea ± SD) (*P *< 0.001). However, the restrictive group received a significantly greater amount of colloid (1, 216 ± 814 ml) than the conventional group (915 ± 559 ml) (*P *< 0.05). The mean doses of dobutamine administered to the conventional and restrictive groups, respectively, were 12.3 ± 7.3 μg/kg/minute vs 10.9 ± 5.9 μg/kg/minute intraoperatively (*P *= 0.32) and 9.5 ± 6.2 vs 8.0 ± 4.5 μg/kg/minute postoperatively (*P *= 0.19). There were 26 achievers (60.4%) in the conventional group and 18 (40%) in the restrictive group during surgery (*P *= 0.089). After surgery, a larger number of patients in both groups achieved the therapeutic goals (31 (73.0%) vs 28 (62.2%); *P *= 0.39).

**Table 2:** Baseline characteristics of patients in the conventional and restrictive groups

Patient characteristics	Conventional group	Restrictive group
Number of patients	43	45
Males (%)	24 (55.8)	21 (47.0)
Age, years	68.6 ± 7.3	69.2 ± 9.0
Cancer	29 (67.4)	33 (73.3)
Risk score, points	4 [3 to 4]	3 [3 to 4]
P-POSSUM physiological score	24 [20 to 27]	22 [18 to 27]
P-POSSUM operative score	15 [11 to 17]	15 [14 to 15]
Predicted morbidity rate	61.5 [52.7 to 82.3]	61.7 [45.7 to 82.4]
Predicted mortality rate	14.6 [11.2 to 38.2]	14.5 [9.1 to 28.9]
Clinical predictors		
Age > 60 years	38 (88.3)	40 (88.8)
Arterial hypertension (difficult to control)	27 (62.7)	27 (60.0)
COPD	4 (9.3)	4 (8.8)
Diabetes	4 (9.3)	4 (8.8)
EKG alterations	4 (9.3)	4 (8.8)
Previous AMI	1 (2.3)	3 (6.6)
Previous CVA	2 (4.6)	0 (0)
Type of surgery		
Colorectal	30 (69.7)	38 (84.4)
Vascular	10 (23.2)	4 (8.8)
Orthopedic	2 (4.6)	2 (4.4)
Other	1 (2.3)	1 (2.2)

AMI = acute myocardial infarction; COPD = chronic obstructive pulmonary disease; CVA = cardiovascular accident; EKG = electrocardiogram; P-POSSUM = Portsmouth predictor equation-modified Physiological and Operative Severity Score for the enUmeration of Mortality and morbidity; SD = standard deviation. Data a presented as *n *(%), mean ± SD or median [25% to 75% interquartile range]. There were no significant differences between groups.

**Table 3:** Therapeutic interventions and changes in perfusion variables in both groups

Variables	Conventional	Restrictive
**Therapeutic intervention**		
Intraoperative		
Operation time, minutes	228 ± 53	250 ± 60
Crystalloid, ml	4, 335 ± 1, 546	2, 301 ± 1, 064**
Colloid, ml	915 ± 559	1, 216 ± 814*
Fluid-challenged patients	42 (97.7)	44 (97.7)
Fluid challenges per patient	2.4	3.1
Positive fluid challenges	61 (58.6)	93 (65.0)
Transfused patients (RBCs)	18 (41.8)	19 (42.2)
RBCs, units	1.8 ± 0.4	1.9 ± 0.9
Dobutamine doses, μg/kg/minute	12.3 ± 7.3	10.9 ± 5.9
Achievers	26 (60.4)	18 (40.0)
Postoperative		
Crystalloid, ml	1, 296 ± 1114	1, 145 ± 680
Colloid, ml	1, 321 ± 595	1, 210 ± 700
RBC transfusions	10 (23.2)	11 (24.4)
RBCs, units	1.7 ± 0.7	1.5 ± 0.5
Dobutamine dose, μg/kg/minute	9.5 ± 6.2	8.0 ± 4.5
**Tissue perfusion**		
Preoperative		
Serum lactate, mEq/L	1.3 ± 0.4	1.3 ± 0.5
S_CV_O_2_, %	72 ± 7	71 ± 7
pH	7.39 ± 0.05	7.41 ± 0.04
ICU admission		
Serum lactate, mEq/L	2.51 ± 1.1	2.6 ± 1.2
S_CV_O_2_, %	72 ± 10	74 ± 10
pH	7.32 ± 0.06	7.32 ± 0.07
24 hours after ICU admission		
Serum lactate, mEq/L	1.9 ± 0.8	2.0 ± 0.9
S_CV_O_2_, %	76 ± 8	72 ± 9*
pH	7.39 ± 0.05	7.3 5 ± 0.05
Achievers	31 (73.0)	28 (62.2)

RBC = red blood cell; S_CV_O_2 _= central venous oxygen saturation; SD = standard deviation. Data are presented as absolute values, *n *(%) or means ± SD. *P < 0.05 vs conventional group. **P < 0.01 vs conventional group.

**Figure 2:**
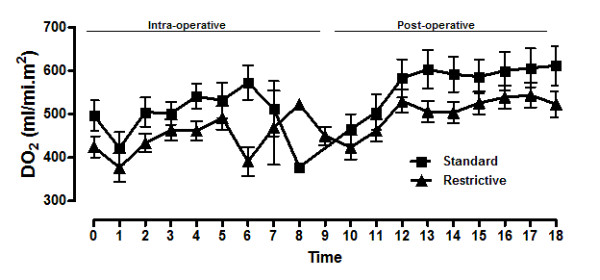
**Temporal patterns**. Temporal patterns of oxygen delivery (DO_2_) during surgery and postoperatively for the restrictive group (black triangles) and the conventional group (black squares). The results are presented as means and standard errors of the mean. 0, preoperative; 1, 30 minutes intraoperatively (IO); 2, 60 minutes IO; 3, 120 minutes IO; 4, 180 minutes IO; 5, 240 minutes IO; 6, 300 minutes IO; 7, 360 minutes IO; 8, 420 minutes IO; 9, 480 minutes IO; 10, ICU admission; 11, 1 hour postoperatively (PO); 12, 2 hours PO; 13, 3 hours PO; 14, 4 hours PO; 15, 5 hours PO; 16, 6 hours PO; 17, 7 hours PO; 18, 8 hours PO. Treatment × time interaction; *P *< 0.0001.

Temporal patterns of DO_2_I were similar between the two groups. After an initial decline following anesthesia induction, DO_2_I recovered similarly in both groups (Figure 2). The *P *value for the treatment vs time interaction was < 0.0001. DO_2_I levels were maintained at levels higher than baseline most of the time in both groups. Patterns of DO_2_I were also similar in patients with or without complications, regardless of group assignment (data not shown). Serum lactate and pH did not differ between groups at baseline, ICU admission or 24 hours after surgery (Table 3). S_CV_O_2 _values were similar at baseline and ICU admission but significantly lower in the restrictive group (72 ± 9) than in the conventional group (76 ± 8) after 24 hours (*P *< 0.05).

All but one patient in each group received at least one fluid challenge. In the conventional group, 104 fluid challenges were performed, of which 58.6% were positive. In the restrictive group, 143 fluid challenges were performed, of which 65% were positive (Table 3).

There were no differences in pulmonary or renal function between groups at any time (Table 4). Serum levels of CRP for the conventional and restrictive groups were 2.0 ± 2.5 mg/dl and 2.1 ± 2.4 mg/dl after admission to the ICU, respectively (*P *= 0.97). Twenty-four hours after admission to the ICU CRP levels were 12.0 ± 5.5 mg/dl in the conventional group and 11.9 ± 6.5 mg/dl in the restrictive group (*P *= 0.94).

**Table 4:** Renal and pulmonary function in conventional and restrictive groups

Renal and pulmonary function	Conventional	Restrictive
Before surgery		
Creatinine, mg/dl	1.0 ± 0.4	0.9 ± 0.3
PO_2_/FiO_2 _ratio	438 ± 220	436 ± 187
ICU admission		
Creatinine, mg/dl	0.9 ± 0.5	1.1 ± 1.2
PO_2_/FiO_2 _ratio	335 ± 174*	337 ± 173*
24 hours after surgery		
Creatinine, mg/dl	1.2 ± 0.7	1.0 ± 0.3
PO_2_/FiO_2 _ratio	260 ± 129**	248 ± 109**

PO_2_/FiO_2 _ratio = ration of arterial oxygen pressure to fraction of inspired oxygen; SD = standard deviation. All data are means ± SD. *P < 0.05 vs before surgery; **P < 0.01 vs before surgery.

In the restrictive group, the rate of major postoperative complications was 52% less than in the conventional group (20.0% vs 41.9%, RR = 0.48, 95% CI = 0.24 to 0.94; *P *= 0.046) (Table 5). The 60-day mortality rates were 6.9% in the conventional group and 2.2% in the restrictive group (not significant).

**Table 5:** Major complications in conventional and restrictive groups

Complications and outcomes	Conventional (*n *= 40)	Restrictive (*n *= 41)
Total complications	43	45
Cardiovascular complications		
Atrial fibrillation	2 (4.6)	1 (2.2)
Hypertensive crisis	4 (9.3)	0 (0)
Pulmonary thromboembolism	0 (0)	1 (2.2)
Total	6 (13.9)	2 (4.4)
Tissue-healing complications		
Evisceration	2 (4.6)	0 (0)
Anastomotic leak	2 (4.6)	1 (2.4)
Total	4 (9.3)	1 (2.4)
Infectious complications		
Nosocomial pneumonia	4 (9.3)	6 (13.3)
Occult septic shock	1 (2.3)	0 (0)
Peritonitis	2 (4.6)	0 (0)
Blood stream infection	0 (0)	1 (2.2)
Wound abscess	2 (4.6)	0 (0)
Total	9	7
Other complications		
Extubation failure	1 (2.3)	0 (0)
Renal dysfunction	1 (2.3)	0 (0)
Gastrointestinal dysfunction	3 (6.9)	1 (2.2)
Total	5	1
Total number of major complications	24	11
Number of patients with complications	18 (41.8)	9 (20.0)*
Number of complications per patient	0.55	0.24
Outcomes		
LOS in the ICU	2.0 [1.0 to 4.0]	2.0 [2.0 to 5.0]
LOS in the hospital	6.0 [4.0 to 9.0]	6.0 [4 to 10]
30-day mortality rate	2 (4.6)	0 (0)
60-day mortality rate	3 (6.9)	1 (2.2)

LOS = length of stay. Data are presented as absolute values, *n *(%) or medians [25% to 75% interquartile range].

## Discussion

This study is the first in which the effects of two different regimens of fluid maintenance in the setting of GDT and optimization of DO_2 _have been investigated. The main finding of our study is that there were significantly fewer major complications, particularly with regard to tissue healing and cardiovascular events, in high-risk patients managed with GDT and a more restrictive fluid strategy during major surgery, compared to those managed with a more liberal fluid strategy. Infusion of 4 ml/kg/hour compared to 12 ml/kg/hour of lactated Ringer's solution as maintenance fluid during GDT with DO_2_- and lactate-guided optimization reduced the incidence of major complications by 52%.

Fluid maintenance during prolonged surgery results in the administration of large amounts of crystalloid, causing weight increases of 3 to 6 kg [22]. There is a dose-response relationship between complications and increasing body weight on the day of surgery. Positive fluid balance has been associated with more complications and increased mortality in medical and surgical patients admitted to ICUs [34-36].

Interventional studies have compared restrictive regimens of fluid maintenance using more liberal strategies. In a randomized controlled study of 172 patients undergoing colorectal surgery, Brandstrup *et al*. [22] showed that not replacing fluid loss to the third space and not giving a volume preload prior to epidural anesthesia was associated with a significant decrease from 56% to 30% in postoperative morbidity. In this study, patients in the restrictive group received 3, 200 ml of fluid compared to 6, 200 ml in the liberal group [22]. Nisanevich *et al*. [23] compared 4 ml/kg/hour with 12 ml/kg/hour of fluid maintenance during gastrointestinal surgery and reported a similar impact on outcomes. In another study, administration of a median of 3, 000 ml compared to 6, 300 ml of fluid reduced complications and length of stay in the hospital after colorectal surgery [26]. Restrictive fluid regimens have also been associated with better outcomes in patients undergoing vascular surgery [28,29].

Our study corroborates the findings of these previous publications. It is important to note, however, that, in contrast to our study, these earlier studies were performed in low-risk patients and GDT was not applied simultaneously with surgery. One recent publication compared two strategies of fluid maintenance, 6 ml/kg/hour of crystalloid in the restrictive group and 12 ml/kg/hour of crystalloid in the conventional group, integrated with GDT in patients undergoing major surgery [36]. In both groups in that study, a fluid bolus was administered when respiratory variation in peak aortic flow velocity was > 13%, which was considered indicative of hypovolemia. The incidence of hypovolemia and postoperative complications, especially anastomotic leak and sepsis, was higher in the restrictive group than in the conventional group. Although these investigators used fluid GDT based on maximization of a flow-related parameter, DO_2 _was not a target, making direct comparisons with our study difficult.

There are two fundamental aspects of GDT in high-risk surgical patients that must be considered. First, hypovolemia is very common in surgical patients and cannot be detected by routine monitoring of blood pressure, heart rate and central venous pressure. Reduced effective circulating volume may cause various deleterious effects by redirecting flow away from the gut, kidneys and other organs, leading to MOF. At the other extreme, fluid overload in critically ill patients has been associated with various adverse effects, such as prolonged mechanical ventilation, pulmonary edema, abdominal compartment syndrome, infection, longer stay in the ICU and more complications after diverse types of surgery. Therefore, one important component of GDT is to test fluid responsiveness with fluid challenges to minimize the risk of hypo- or hypervolemia. Fluid responders are able to convert fluid loading into a significant increase in CO.

Many studies of GDT have used successive fluid challenges as the main tool of the GDT strategy and have shown improved outcomes mainly as decreased complication rates [12-20]. In older patients, however, fluid challenge unresponsiveness is not infrequently related to myocardial dysfunction or intolerance to fluids. In our study, all but one patient in each group had signs of hypovolemia during surgery as defined by the treatment algorithm. Patients in the restrictive group received more fluid challenges (3.1 per patient) than did patients in the conventional group (2.4 per patient). However, the number of fluid responders was similar between the two groups, demonstrating that although patients in the conventional group received more crystalloid, it did not prevent further signs of hypovolemia, which is likely a reflection of cardiac dysfunction.

A second important aspect of GDT in high-risk patients is to prevent an imbalance between DO_2 _and VO_2 _to avoid multiple organ dysfunction. Many researchers have used dobutamine to achieve higher levels of DO_2_I or cardiac index during prolonged surgery and have reported reduced morbidity and mortality [6-11]. One key difference between our study and the study by Futier *et al*. [37], apart from the different goal of therapy, is the use of dobutamine. The fact that in our study there were fewer fluid responders suggests that our population had more contractility problems, which is consistent with the older median age of our group. Dobutamine may increase tolerance to fluids and prevent cardiac dysfunction [33]. These data suggest that dobutamine may be important in this group of patients and that DO_2 _may be a more appropriate goal for hemodynamic optimization therapy in high-risk patients.

Edema of the intestines and other tissues may be responsible for poor tissue healing and other complications. Gut edema may be associated with postoperative gastrointestinal dysfunction, impaired tissue oxygenation and increased intraabdominal pressure. The use of less crystalloid associated with more colloid, by protecting the gut from gastrointestinal dysfunction, may explain the reduced complication rate in the restrictive group. In experimental models, colloid increases microcirculatory blood flow and tissue oxygen tension in the gut mucosa [38]. In a model of gut ischemia in rabbits, crystalloid administration was associated with more gut edema than a combination of colloid with less crystalloid [39]. Patients in the restrictive group received more fluid challenges (3.1 per patient) than did patients in the conventional group (2.4 per patient). As a consequence, they received more colloid. It is possible that the fluid challenges rather than the overall fluid balance benefit patients the most.

It is now recognized that around 15% of patients undergoing surgery are at high risk of complications and death [40]. Factors associated with increased risk include older age, the presence of comorbid disease and major surgical procedures. In the present study, patients had a median of four comorbidities and a median age of 70 years. Researchers in previous studies have published morbidity rates as high as 70% in similar groups of patients. A previous study in Brazilian ICUs reported morbidity and mortality rates of 38% and 20%, respectively, in unselected surgical patients [4]. Major complications occurred in 32% of patients in an Australian study with a mortality rate of 2% [41]. The low morbidity and mortality, despite higher rates predicted on the basis of POSSUM scores, suggests that the preemptive use of GDT with fluids and inotropes targeting DO_2 _improves survival in this group of surgical patients.

Our study has strengths and limitations. First, the volume used as maintenance fluid may be considered too liberal. We deliberately chose a conventional fluid regimen that reflects current clinical practice for fluid administration during major surgical procedures. Textbooks and guidelines indicate a need for 10 to 15 ml/kg/hour of crystalloids as maintenance fluid in addition to the replacement of blood loss during major and prolonged surgery. The basis for this standard recommendation is an assumed large intravascular volume deficit caused by evaporative loss, fasting and third spacing, all to be replaced by crystalloids. More recent studies have actually shown that extracellular volume expands rather than contracts with fluid balance [42]. Despite earlier studies and several clinical guidelines suggesting benefits associated with a more restrictive or net even approach to fluid therapy, this strategy is still not widely used. Second, this study was performed in only one tertiary center, and its results may not be applicable to other centers. Third, the inability to include consecutive patients because of the limited number of anesthesiologists trained in the procedures used in the study is also a limitation. The main strength of our study is its randomized controlled design and the homogeneous group of high-risk surgical patients.

## Conclusions

Perioperative hemodynamic GDT with a protocol incorporating restrictive fluid maintenance and inotropic therapy to achieve the best possible DO_2 _in very high-risk surgical patients can be easily performed with the use of minimally invasive hemodynamic monitoring to obtain continuous monitoring of CO and is related to better patient outcomes. It is possible that with more reliable and easy-to-use hemodynamic monitoring during anesthesia, fluid maintenance volumes might be safely reduced.

## Key messages

◆ Morbidity and mortality are high in some groups of patients, particularly elderly patients with low cardiorespiratory reserves who undergo extensive noncardiac surgery.

◆ Perioperative alterations in DO_2 _are closely correlated to the development of MOF and death.

◆ Randomized clinical trials have shown reductions in postoperative complications and mortality rates when strategies aimed at optimizing tissue perfusion are used in the perioperative period.

◆ The optimal perioperative fluid regimen for major noncardiac surgery patients is unclear.

◆ The preemptive use of GDT with fluids and inotropes targeting DO_2 _and a more restrictive strategy of fluid maintenance during surgery leads to fewer major complications in high-risk patients undergoing major surgery.

## Competing interests

The authors declare that they have no competing interests.

## Authors' contributions

SML contributed to the study design, the statistical analysis, the analysis and interpretation of results, the drafting of the manuscript and critical revisions of the manuscript. LSR contributed to the study design and the acquisition, analysis and interpretation of data. KN was the study coordinator. FRML contributed to the study design, acquisition of data, conduct of the trial, analysis and interpretation of the results and critical revision of the manuscript. NEO and GSC contributed to the study design, acquisition of data and conduct of the trial. PGB contributed to the acquisition of data, the conduct of the trial and the analysis and interpretation of results. AF contributed to the acquisition of data and the conduct of the trial. JGN contributed to the study design and acquisition of data.
